# Complete genome sequence of *Saccharomonospora viridis* type strain (P101^T^)

**DOI:** 10.4056/sigs.20263

**Published:** 2009-09-24

**Authors:** Amrita Pati, Johannes Sikorski, Matt Nolan, Alla Lapidus, Alex Copeland, Tijana Glavina Del Rio, Susan Lucas, Feng Chen, Hope Tice, Sam Pitluck, Jan-Fang Cheng, Olga Chertkov, Thomas Brettin, Cliff Han, John C. Detter, Cheryl Kuske, David Bruce, Lynne Goodwin, Patrick Chain, Patrik D'haeseleer, Amy Chen, Krishna Palaniappan, Natalia Ivanova, Konstantinos Mavromatis, Natalia Mikhailova, Manfred Rohde, Brian J. Tindall, Markus Göker, Jim Bristow, Jonathan A. Eisen, Victor Markowitz, Philip Hugenholtz, Nikos C. Kyrpides, Hans-Peter Klenk

**Affiliations:** 1 DOE Joint Genome Institute, Walnut Creek, California, USA; 2 DSMZ - German Collection of Microorganisms and Cell Cultures GmbH, Braunschweig, Germany; 3 Los Alamos National Laboratory, Bioscience Division, Los Alamos, New Mexico, USA; 4 Lawrence Livermore National Laboratory, Livermore, California, USA; 5 Biological Data Management and Technology Center, Lawrence Berkeley National Laboratory, Berkeley, California, USA; 6 HZI - Helmholtz Centre for Infection Research, Braunschweig, Germany; 7 University of California Davis Genome Center, Davis, California, USA

**Keywords:** thermophile, hot compost, Gram-negative actinomycete, farmer’s lung disease, bagassosis, humidifier fever, pentachlorophenol metabolism, *Pseudonocardiaceae*

## Abstract

*Saccharomonospora viridis* (Schuurmans *et al.* 1956) Nonomurea and Ohara 1971 is the type species of the genus *Saccharomonospora* which belongs to the family *Pseudonocardiaceae*. *S. viridis* is of interest because it is a Gram-negative organism classified among the usually Gram-positive actinomycetes. Members of the species are frequently found in hot compost and hay, and its spores can cause farmer’s lung disease, bagassosis, and humidifier fever. Strains of the species *S. viridis* have been found to metabolize the xenobiotic pentachlorophenol (PCP). The strain described in this study has been isolated from peat-bog in Ireland. Here we describe the features of this organism, together with the complete genome sequence, and annotation. This is the first complete genome sequence of the family *Pseudonocardiaceae*, and the 4,308,349 bp long single replicon genome with its 3906 protein-coding and 64 RNA genes is part of the *** G****enomic* *** E****ncyclopedia of* *** B****acteria and* *** A****rchaea * project.

## Introduction

Strain P101^T^ (= DSM 43017 = ATCC 15386 = JCM 3036 = NCIMB 9602) is the type strain of *Saccharomonospora viridis*, and the type species of the genus *Saccharomonospora* [1,2], which currently contains eight species [3]. Although phylogenetically a member of the Gram-positive actinomycetes, already the initial report on *S. viridis* strain P101^T^ noticed the astonishing feature of the organism to be Gram-negative, despite showing the typical mycelium morphology of *Saccharomonospora* [2]. Like in other actinomycetes, spores of *S. viridis* are readily dispersed in air, and the prolonged exposure to spores can apparently result in acute respiratory distress (farmer’s lung disease) which may lead to irreversible lung damage [4,5]. Here we present a summary classification and a set of features for *S. viridis* P101^T^, together with the description of the complete genomic sequencing and annotation.

## Classification and features

Members of the species *S. viridis* have been isolated or molecularly identified on several occasions from hot composts in Europe and USA [12-14,17], and also from soil in Japan [1]. One novel, yet unpublished, cultivated member of the species has been reported by Lu and Liu from Chinese soil (AF127525). Uncultured clone sequences with significant (99%) sequence similarity were observed from composting mass in China (AM930281 and AM930338). Screening of environmental genomic samples and surveys reported at the NCBI BLAST server indicated no closely related phylotypes that can be linked to the species or genus, with the closest matches (about 90% sequence similarity) to strain P101^T^ 16S rRNA identified in a marine metagenome from the Sargasso Sea [18].

**Figure 1:**
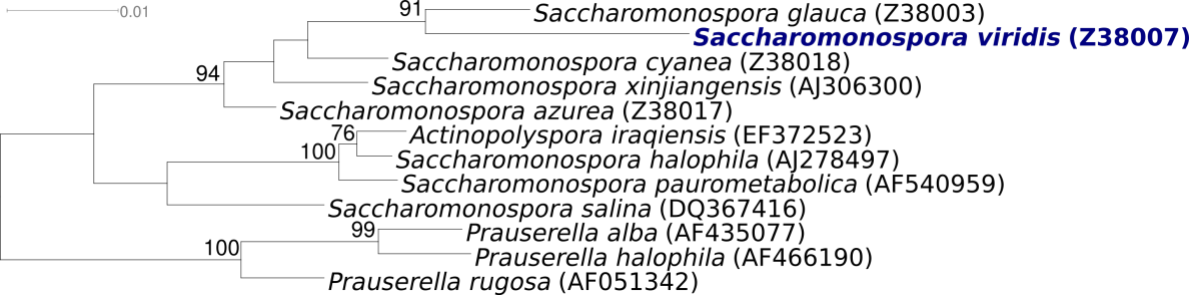
Phylogenetic tree of *S. viridis* strain P101^T^ and all type strains of the genus *Saccharomonospora* inferred from 1,474 aligned characters [19,20] of the 16S rRNA gene under the maximum likelihood criterion [21]. The tree was rooted with all type strains of the members of the genus *Prauserella*, another genus in the family *Pseudonocardiaceae*. The branches are scaled in terms of the expected number of substitutions per site. Numbers above branches are support values from 1,000 bootstrap replicates if larger than 60%. Lineages with type strain genome sequencing projects registered in GOLD [22] are shown in blue, published genomes in bold.

Figure 1 shows the phylogenetic neighborhood of *S. viridis* strain P101^T^ in a 16S rRNA based tree. The sequences of all three copies of the 16S rRNA gene are identical and perfectly match the previously published 16S rRNA sequence generated from NCIMB 9602 (Z38007).

**Figure 2:**
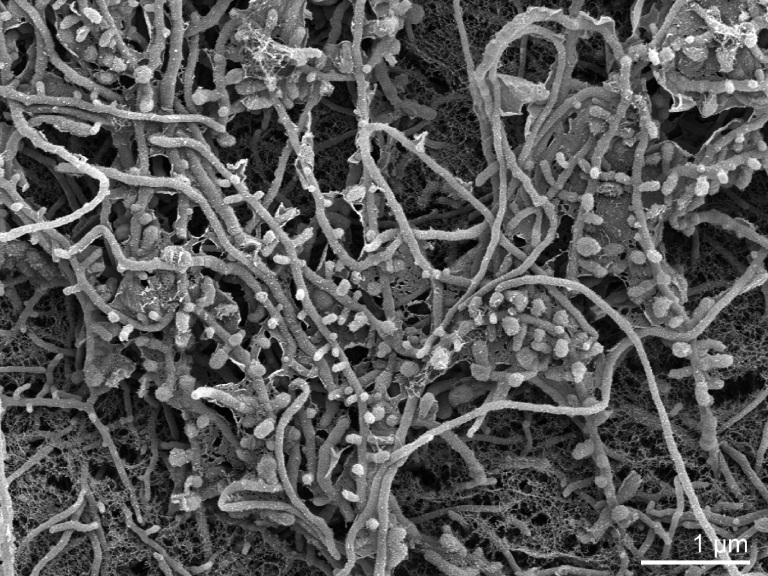
Scanning electron micrograph of *S. viridis* P101^T^

The hyphae of the vegetative mycelium of strain P101^T^ are branched and sometimes show curved endings [12]. Single spores are observed only on the aerial mycelium either directly on the hyphae or on short sporophores (Table 1 and Figure 2). The spores are oval, 0.9-1.1 µm × 1.2-1.4 µm in size. Only very occasionally two spores are observed. The aerial mycelium is either grayish green in color, or turns from white to greenish as on Czapek Agar. The optimal temperature for growth is 55°C, but 45°C for aerial mycelium formation and pigment production. At 37°C and 60°C the growth is very limited and without aerial mycelia. No growth occurs at 27°C and 70°C [12].

**Table 1:** Classification and general features of *S. viridis* P101^T^ in accordance with the MIGS recommendations [6]

**MIGS ID**	**Property**	**Term**	**Evidence code**
	Current classification	Domain *Bacteria*	TAS [7]
		Phylum *Actinobacteria*	TAS [8]
		Order *Actinomycetales*	TAS [9]
		Suborder *Pseudonocardineae*	TAS [9]
		Family *Pseudonocardiaceae*	TAS [9]
		Genus *Saccharomonospora*	TAS [1]
		Species *Saccharomonospora viridis*	TAS [2]
		Type strain P101	
	Gram stain	negative	TAS [2]
	Cell shape	variable	TAS [10]
	Motility	nonmotile	NAS
	Sporulation	single spores mainly on aerial mycelium	TAS [1]
	Temperature range	thermophile, 37-60°C	TAS [11]
	Optimum temperature	55°C for growth, 45°C for aerial mycelium formation	TAS [1,11,12]
	Salinity	7% NaCl	TAS [11]
MIGS-22	Oxygen requirement	aerobic; nor reported if essential	TAS [11]
	Carbon source	D-glucose, sucrose, dextrin	TAS [11]
	Energy source	carbohydrates	TAS [11]
MIGS-6	Habitat	peat and compost (species occurrence)	TAS [1,4,12-14]
MIGS-15	Biotic relationship	free living	
MIGS-14	Pathogenicity	lung damage	TAS [4]
	Biosafety level	1	TAS [15]
	Isolation	peat-bog at 250 cm depth	TAS [12]
MIGS-4	Geographic location	Irish peat	
MIGS-5	Sample collection time	before 1963	TAS [12]
MIGS-4.1 MIGS-4.2	Latitude – Longitude	not reported	
MIGS-4.3	Depth	not reported	
MIGS-4.4	Altitude	not reported	

Evidence codes - IDA: Inferred from Direct Assay (first time in publication); TAS: Traceable Author Statement (i.e., a direct report exists in the literature); NAS: Non-traceable Author Statement (i.e., not directly observed for the living, isolated sample, but based on a generally accepted property for the species, or anecdotal evidence). These evidence codes are from the Gene Ontology project [16]. If the evidence code is IDA, then the property was observed for a living isolate by one of the authors, or an expert mentioned in the acknowledgements.

Strain P101^T^ has been observed to be sensitive to a variety of phages [11]. Members of *S. viridis* are apparently able to metabolize pentachlorophenol but not other chlorophenols [14]. It was suggested that *S. viridis* metabolizes PCP by conjugation to form a more polar transformation product, but, unlike other PCP-degrading bacteria, the organism is incapable of effecting total degradation of the xenobiotic [14]. Microorganisms such as *S. viridis* may therefore contribute to PCP removal by microbial communities in situ, despite being unable to completely mineralize chlorophenols in pure culture [14]. *S. viridis* produces a thermostable α-amylase which forms 63% (w/w) maltose on hydrolysis of starch [23]. Maltotriose and maltotetraose are the only intermediate products observed during this reaction, with maltotriose accumulating to 40% (w/w). Both unimolecular and multimolecular mechanisms (transfers and condensation) have been shown to occur during the concentration-dependent degradation of maltotriose and maltotetraose. Such reactions result in the almost exclusive formation of maltose from maltotriose at high initial concentration [23]. *S. viridis* produces thermoviridin, an antibiotic that is primarily active against the Gram-positive bacteria (growth inhibition) [2,11]. At higher concentrations, also Gram-negative bacteria were growth-inhibited [2].

### Chemotaxonomy

The murein of P101^T^ is of cell wall type IV. It contains meso-diaminopimelic acid in the peptidoglycan and arabinose and galactose in whole-cell hydrolysates (sugar type A). Mycolic acids and teichonic acids were not reported. Strain P101^T^ contains menaquinones MK-9(H_4_) (60%) and MK-8(H_4_) (20 to 30%). The combination of the tetrahydromultiprenyl menaquinones MK-9(H_4_) and MK-8(H_4_) is characteristic for the genus *Saccharomonospora* [11]. The major cellular fatty acids are saturated, iso-branched acids with 16 and 18 carbon atoms, and 2-hydroxydodecanoic acids. Details are described in the Compendium of *Actinobacteria* [10]. Phosphatidylethanolamine, hydroxy-phosphatidyl-ethanolamine, and lyso-phosphatidyl-ethanolamine were identified as the main phospholipids.

## Genome sequencing and annotation

### Genome project history

This organism was selected for sequencing on the basis of its phylogenetic position, and is part of the *** G****enomic* *** E****ncyclopedia of* *** B****acteria and* *** A****rchaea * project. The genome project is deposited in the Genome OnLine Database [22] and the complete genome sequence in GenBank. Sequencing, finishing and annotation were performed by the DOE Joint Genome Institute (JGI). A summary of the project information is shown in Table 2.

**Table 2:** Genome sequencing project information

**MIGS ID**	**Property**	**Term**
MIGS-31	Finishing quality	Finished
MIGS-28	Libraries used	Two Sanger libraries - 8 kb pMCL200 and fosmid pcc1Fos
MIGS-29	Sequencing platforms	ABI3730
MIGS-31.2	Sequencing coverage	12.9 Sanger
MIGS-30	Assemblers	phrap
MIGS-32	Gene calling method	Genemark 4.6b, tRNAScan-SE-1.23, infernal 0.81, GenePRIMP
	INSDC / Genbank ID	CP001683
	Genbank Date of Release	August 26, 2009
	GOLD ID	Gc01088
	NCBI project ID	20835
	Database: IMG-GEBA	2500901760
MIGS-13	Source material identifier	DSM 43017
	Project relevance	Tree of Life, GEBA

### Growth conditions and DNA isolation

*S. viridis* strain P101^T^, DSM 43017, was grown in DSMZ medium 535 (Trypticase soy broth, ) at 45°C. DNA was isolated from 1-1.5 g of cell paste using Qiagen Genomic 500 DNA Kit (Qiagen, Hilden, Germany) with a modified protocol, st/FT, for cell lysis, as described in Wu *et al*. [24].

### Genome sequencing and assembly

The genome was sequenced using Sanger sequencing platform only. All general aspects of library construction and sequencing can be found at the JGI website (http://www.jgi.doe.gov). The Phred/Phrap/Consed software package was used for sequence assembly and quality assessment. After the shotgun stage reads were assembled with parallel phrap (High Performance Software, LLC). Possible mis-assemblies were corrected with Dupfinisher [25] or transposon bombing of bridging clones (Epicentre Biotechnologies, Madison, WI). Gaps between contigs were closed by editing in Consed, custom primer walk or PCR amplification (Roche Applied Science, Indianapolis, IN). A total of 354 finishing reactions were produced to close gaps and to raise the quality of the finished sequence. The completed genome sequences of *S. viridis* contains 66,210 Sanger reads, achieving an average of 12.9 sequence coverage per base, with an error rate less than 1 in 100,000.

### Genome annotation

Genes were identified using GeneMark [26] as part of the genome annotation pipeline in the Integrated Microbial Genomes Expert Review (IMG-ER) system [27], followed by a round of manual curation using the JGI GenePRIMP pipeline (http://geneprimp.jgi-psf.org) [28]. The predicted CDSs were translated and used to search the National Center for Biotechnology Information (NCBI) nonredundant database, UniProt, TIGRFam, Pfam, PRIAM, KEGG, COG, and InterPro databases. The tRNAScanSE tool [29] was used to find tRNA genes, whereas ribosomal RNAs were found by using the tool RNAmmer [30]. Other non coding RNAs were identified by searching the genome for the Rfam profiles using INFERNAL (v0.81) [31]. Additional gene prediction analysis and manual functional annotation was performed within the Integrated Microbial Genomes (IMG) platform (http://img.jgi.doe.ogv/er) [32].

### Metabolic network analysis

The metabolic Pathway/Genome Database (PGDB) was computationally generated using Pathway Tools software version 12.5 [33] and MetaCyc version 12.5 [34], based on annotated EC numbers and a customized enzyme name mapping file. It has undergone no subsequent manual curation and may contain errors, similar to a Tier 3 BioCyc PGDB [35].

### Genome properties

**Figure 3:**
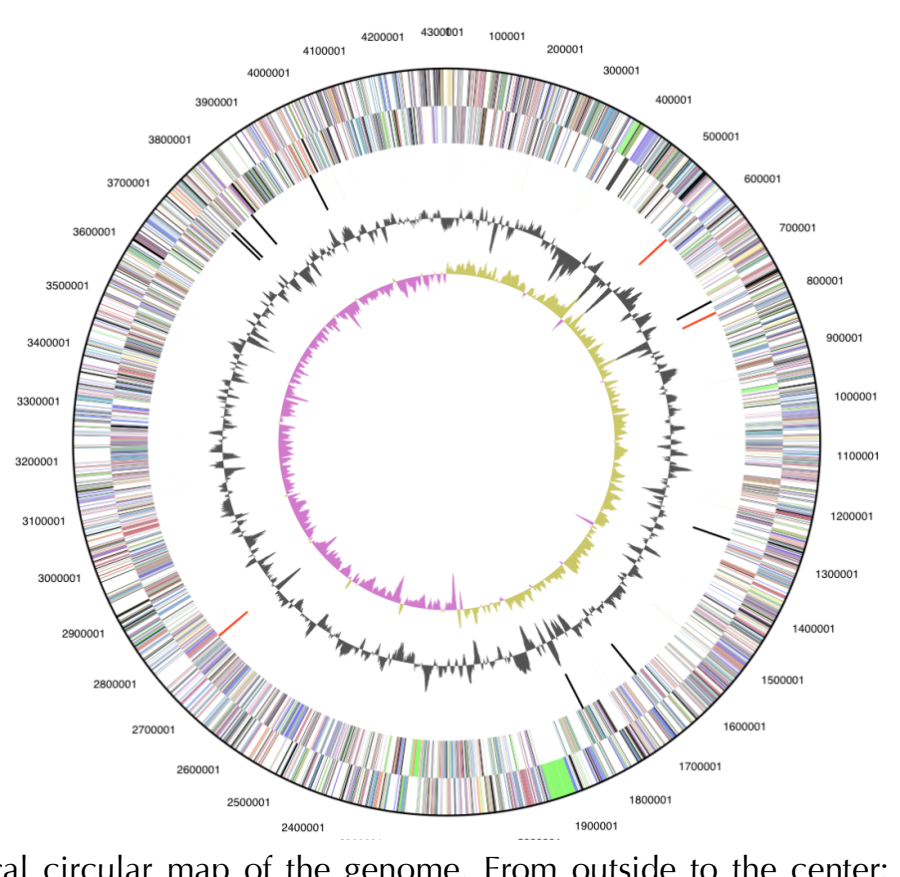
Graphical circular map of the genome. From outside to the center: Genes on forward strand (color by COG categories), Genes on reverse strand (color by COG categories), RNA genes (tRNAs green, rRNAs red, other RNAs black), GC content, GC skew.

**Figure 4:**
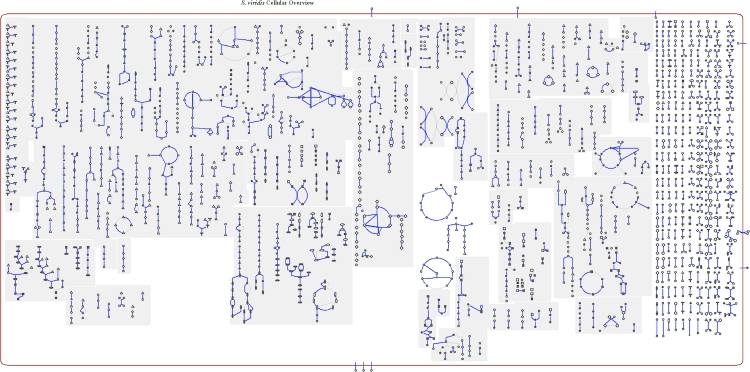
Cellular overview diagram. This diagram provides a schematic of all pathways of *S. viridis* strain P101^T^ metabolism. Nodes represent metabolites, with shape indicating class of metabolite (see key to right). Lines represent reactions.

The genome is 4,308,349 bp long and comprises one main circular chromosome with a 67.3% GC content (Table 3 and Figure 3). Of the 3,970 genes predicted, 3,906 were protein coding genes, and 64 RNAs; 78 pseudogenes were also identified. The majority of the protein-coding genes (71.2%) were assigned with a putative function, while the remaining ones were annotated as having hypothetical function. The properties and the statistics of the genome are summarized in Table 3. The distribution of genes into COGs functional categories is presented in Table 4 and a cellular overview diagram is presented in Figure 4, followed by a summary of metabolic network statistics shown in Table 5.

**Table 3:** Genome Statistics

**Attribute**	**Value**	**% of Total**
Genome size (bp)	4,308,349	100.00%
DNA Coding region (bp)	3,805,483	88.33%
DNA G+C content (bp)	2,900,171	67.32%
Number of replicons	1	
Extrachromosomal elements	0	
Total genes	3,970	100%
RNA genes	64	1.61%
rRNA operons	3	
Protein-coding genes	3,906	98.39%
Pseudo genes	78	1.96%
Genes with function prediction	2,828	71.23%
Genes in paralog clusters	534	13.45%
Genes assigned to COGs	2,709	68.24%
Genes assigned Pfam domains	2,845	71.66%
Genes with signal peptides	725	18.26%
Genes with transmembrane helices	880	22.17%
CRISPR repeats	9	

**Table 4:** Number of genes associated with the general COG functional categories

**Code**	**Value**	**%**	**Description**
J	158	4.0	Translation, ribosomal structure and biogenesis
A	1	0.0	RNA processing and modification
K	276	7.1	Transcription
L	125	3.2	Replication, recombination and repair
B	1	0.0	Chromatin structure and dynamics
D	25	0.6	Cell cycle control, mitosis and meiosis
Y	0	0.0	Nuclear structure
V	44	1.1	Defense mechanisms
T	146	3.7	Signal transduction mechanisms
M	125	3.2	Cell wall/membrane biogenesis
N	2	0.1	Cell motility
Z	0	0.0	Cytoskeleton
W	0	0.0	Extracellular structures
U	27	0.7	Intracellular trafficking and secretion
O	107	2.7	Posttranslational modification, protein turnover, chaperones
C	214	5.5	Energy production and conversion
G	214	5.5	Carbohydrate transport and metabolism
E	293	7.5	Amino acid transport and metabolism
F	85	2.2	Nucleotide transport and metabolism
H	175	4.5	Coenzyme transport and metabolism
I	189	4.8	Lipid transport and metabolism
P	146	3.7	Inorganic ion transport and metabolism
Q	139	3.6	Secondary metabolites biosynthesis, transport and catabolism
R	389	10.0	General function prediction only
S	182	4.7	Function unknown
-	1197	30.6	Not in COGs

**Table 5:** Metabolic Network Statistics

**Attribute**	Value
Total genes	3,970
Enzymes	880
Enzymatic reactions	1,155
Metabolic pathways	244
Metabolites	863
